# A patient-centered deprescribing intervention for hospitalized older patients with polypharmacy: rationale and design of the Shed-MEDS randomized controlled trial

**DOI:** 10.1186/s12913-019-3995-3

**Published:** 2019-03-14

**Authors:** Eduard E. Vasilevskis, Avantika S. Shah, Emily K. Hollingsworth, Matthew S. Shotwell, Amanda S. Mixon, Susan P. Bell, Sunil Kripalani, John F. Schnelle, Sandra F. Simmons, the Shed-MEDS Team, Carole Bartoo, Jennifer Kim, Kanah Lewallen, Whitney Narramore, Robin Parker, Susan Lincoln, Joanna Gupta

**Affiliations:** 1 Vanderbilt University Medical Center, Center for Quality Aging, Nashville, TN USA; 2 Division of Geriatrics, Vanderbilt University Medical Center, Nashville, TN USA; 3 VA Tennessee Valley Healthcare System, Geriatric Research Education and Clinical Center, Nashville, TN USA; 4 Vanderbilt University Medical Center, Section of Hospital Medicine, Nashville, TN USA; 5 Department of Biostatistics, Vanderbilt University, Nashville, TN USA; 6 Division of Cardiovascular Medicine, Vanderbilt University Medical Center, Nashville, TN USA; 7 Vanderbilt University Medical Center, Center for Clinical Quality and Implementation Research, Nashville, TN USA

**Keywords:** Polypharmacy, Deprescribing, Geriatric syndromes, Medications, Geriatrics, Adverse drug events

## Abstract

**Background:**

Polypharmacy is prevalent among hospitalized older adults, particularly those being discharged to a post-care care facility (PAC). The aim of this randomized controlled trial is to determine if a patient-centered deprescribing intervention initiated in the hospital and continued in the PAC setting reduces the total number of medications among older patients.

**Methods:**

The Shed-MEDS study is a 5-year, randomized controlled clinical intervention trial comparing a patient-centered describing intervention with usual care among older (≥50 years) hospitalized patients discharged to PAC, either a skilled nursing facility (SNF) or an inpatient rehabilitation facility (IPR). Patient measurements occur at hospital enrollment, hospital discharge, within 7 days of PAC discharge, and at 60 and 90 days following PAC discharge. Patients are randomized in a permuted block fashion, with block sizes of two to four. The overall effectiveness of the intervention will be evaluated using total medication count as the primary outcome measure. We estimate that 576 patients will enroll in the study. Following attrition due to death or loss to follow-up, 420 patients will contribute measurements at 90 days, which provides 90% power to detect a 30% versus 25% reduction in total medications with an alpha error of 0.05. Secondary outcomes include the number of medications associated with geriatric syndromes, drug burden index, medication adherence, the prevalence and severity of geriatric syndromes and functional health status.

**Discussion:**

The Shed-MEDS trial aims to test the hypothesis that a patient-centered deprescribing intervention initiated in the hospital and continuing through the PAC stay will reduce the total number of medications 90 days following PAC discharge and result in improvements in geriatric syndromes and functional health status. The results of this trial will quantify the health outcomes associated with reducing medications for hospitalized older adults with polypharmacy who are discharged to post-acute care facilities.

**Trial registration:**

This trial was prospectively registered at clinicaltrials.gov (NCT02979353). The trial was first registered on 12/1/2016, with an update on 09/28/17 and 10/12/2018.

**Electronic supplementary material:**

The online version of this article (10.1186/s12913-019-3995-3) contains supplementary material, which is available to authorized users.

## Background

### Polypharmacy and geriatric syndromes

Polypharmacy, defined as taking five or more medications, is common among older patients [1–4]. Studies have shown that approximately 45% of hospitalized older patients are discharged on five or more medications [5–7]. Older patients have an increased prevalence of multi-morbidity; thus, it is not surprising polypharmacy is prevalent. However, a substantial number of medications prescribed to older patients may be unnecessary. More than 90% of inpatients are taking at least one inappropriate medication and up to 43% of medications taken by older patients lack a clear indication. Moreover, 5 to 11% of medications may be unintentionally prescribed for the same indication [8, 9]. Even when a clear indication exists, medications may be inappropriate when considering drug-drug or drug-disease interactions [10, 11]. These medications, also known as potentially inappropriate medications (PIMs), have been defined by multiple explicit criteria such as the Beers list [12], the Screening Tool of Older Persons’ potentially inappropriate Prescriptions (STOPP) [13, 14] and variations of STOPP [15]. Beyond medical appropriateness, some medications may be costly or have inconvenient dosing that decreases patient adherence [8, 9]. Finally, prescribed medications may be inconsistent with a patient’s goals of care [16, 17].

The prevalence and inappropriateness of polypharmacy may lead to multiple harmful outcomes among older community-dwelling and hospitalized populations. These outcomes include, but are not limited to, medication non-adherence [18], adverse drug events [18–20], and increased health care utilization and costs [21–24]. Polypharmacy and a variety of drug indices that quantify drug burden have additionally been associated with the development of geriatric syndromes [25, 26]. Notably, polypharmacy is associated with long-term cognitive impairment [27–29], delirium [30, 31], falls [1, 32–36] frailty [36–38], urinary incontinence [39–41], and unintentional weight loss [42–44].

A recently published study identified specific medications associated with geriatric syndromes [45]. This study showed that hospitalized older patients were prescribed a median of 6 medications per person that could be causing or exacerbating one or more geriatric syndromes. In a study of 904 hospitalized patients aged 65 and older, who were discharged to skilled nursing facilities (SNFs), 57% endorsed three or more geriatric syndromes and 80% endorsed two or more syndromes [46]. The results of this same study showed that 98% of these older inpatients were prescribed 5 or more medications (polypharmacy) and 83% were prescribed 10 or more, sometimes referred to as ‘hyper-polypharmacy’. The average total number of medications at the point of the transition from the hospital to SNF was 13.8 (± 4.9) per patient. Similar to polypharmacy, the presence of multiple geriatric syndromes is predictive of poor health outcomes, even when controlling for age and illness severity [47]. Moreover, patients discharged from the hospital to SNF are at higher risk for loss of independence relative to patients discharged home [48–50]*.*

### Interventions to deprescribe medications and knowledge gaps

In recognition of the potential harms of polypharmacy, numerous studies have evaluated efforts to improve medication prescribing practices for older patients [51–55]. Most interventions have applied the use of explicit criteria to reduce inappropriate prescribing for specific medication classes utilizing tools such as the Beers list or STOPP criteria, while only a few have considered other patient-centered factors (e.g., cost, convenience, life expectancy). Previous trials have commonly used pharmacists, physicians or inter-professional teams to implement deprescribing protocols [52–54]. However, there are several important gaps in our current knowledge. First, few interventions have been initiated in the hospital setting, and no interventions have deprescribed across the continuum of acute and post-acute care [54, 56–59]. Second, few trials have incorporated patient preferences into the decision-making process [60–63]. Finally, although most of the trials reported improvements in medication appropriateness or discrepancies, there has been no study, to date, to evaluate the effects of deprescribing on multiple geriatric syndromes among older patients**.**

The overarching aim of the Shed-MEDS trial is to determine the effects of a hospital-initiated patient-centered deprescribing intervention among older patients discharged to a post-acute care (PAC) facility, either a skilled nursing facility (SNF) or an inpatient rehabilitation facility (IPR). The primary aim is to determine the effect of patient-centered deprescribing on the change in the total number of medications, which includes the number of potentially inappropriate medications (PIMs), number of medications associated with geriatric syndromes (MAGS) and medications that contribute to anticholinergic and sedative drug burden, known as the drug burden index (DBI). Secondary aims of the trial include assessing the effect of the deprescribing intervention on medication adherence, functional health status, and the prevalence and severity of geriatric syndromes. The results of this trial will quantify the health outcomes associated with reducing medications for hospitalized older adults with polypharmacy who are discharged to post-acute care facilities.

## Methods / design

### Protocol reporting

This protocol has been prepared according to the Standard Protocol Items: Recommendations for Interventional Trials (SPIRIT) Statement [64, 65]. Trial results will be reported according to the Consolidated Standards of Reporting Trials (CONSORT) Statement [66, 67]. This trial was registered at clinicaltrials.gov (NCT02979353), initially on 12/1/2016, with an update on 09/28/2017 and 10/12/2018. (ACTRN12617001129370). The SPIRIT Checklist is provided as an Additional file 1 and the SPIRIT Table is included as Table 1.

**Table 1:**
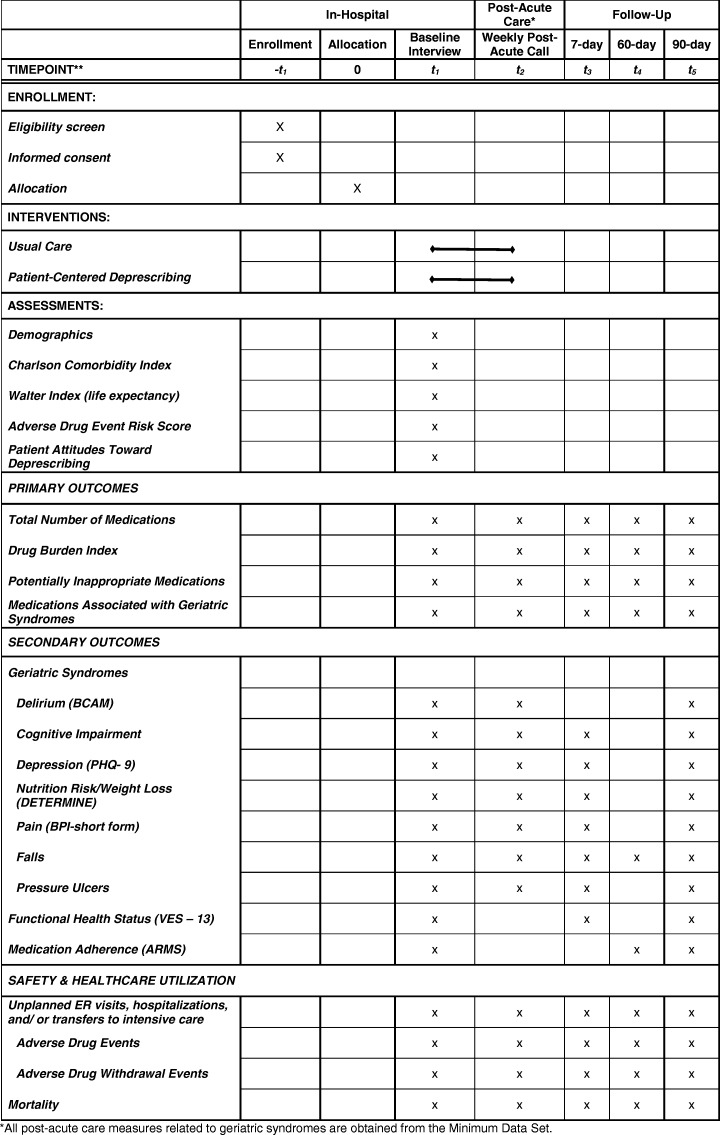
Enrollment, Interventions and Assessments according to the Standard Protocol Items: Recommendations for Intervention Trials (SPIRIT) Diagram. *All post-acute care measures related to geriatric syndromes are obtained from the Minimum Data Set.

### Trial design

**Fig. 1:**
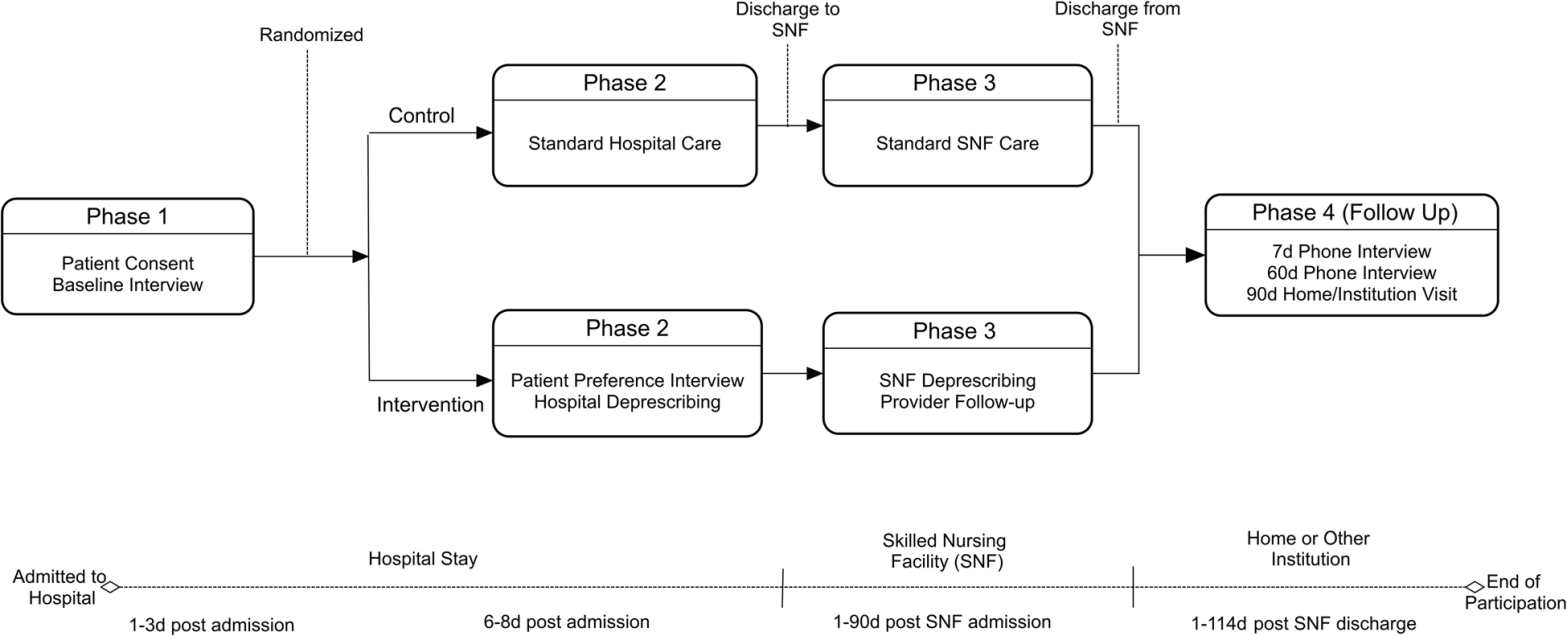
Flow of Participants through Study

Shed-MEDS is a 5-year randomized, controlled, un-blinded clinical trial at a single academic medical center, funded by the National Institute of Aging (NIA). The intervention is not blinded to enrolled patients; however, group assignment (intervention versus control) and safety outcome assessments are blinded to the outcome assessors not engaged in intervention implementation. The trial design is shown in Fig. 1. All study procedures were reviewed and approved by the academic affiliated Institutional Review Board (IRB) and the NIA-appointed Data Safety and Monitoring Board (DSMB). Protocol amendments are first approved by the affiliated IRB then communicated to the NIA and DSMB via the semi-annual DSMB meeting. Other relevant parties are notified through updates to the study information on the clinicaltrials.gov website. All publications from the study will include a summary of all protocol amendments.

### Study setting, participants, and eligibility criteria

Patient enrollment for the Shed-MEDS trial began March, 2017 and is scheduled to end October, 2020. Adults, aged 50 and older, hospitalized at Vanderbilt University Medical Center (VUMC) and referred to post-acute care (PAC) at one of 20 skilled nursing facilities (SNFs) or two inpatient rehabilitation facilities (IPRs) in the Middle Tennessee area are potentially eligible for the study. The age criterion was lowered from 65 to 50 after four months of initial study enrollment because a substantial proportion of hospitalized adults aged 50–64 otherwise met all eligibility criteria. This protocol modification was approved by the IRB. Each of the 22 “partner” PAC facilities (SNF or IPR) provided contact information for designated licensed nursing personnel and a prescribing authority to allow the research team to communicate deprescribing actions for intervention participants and retrieve relevant health information for all study participants during their PAC stay. The written informed consent covers all participating PACs.

Trained research personnel screen patients for additional eligibility criteria on weekdays based on electronic medical record data. Additional study inclusion criteria require the patient to have: five or more medications on their pre-hospital admission medication list (to include all prescription and over-the-counter medications, both scheduled and as needed) and a home residence in one of nine surrounding counties of VUMC to facilitate a home visit during the study follow-up phase 90-days after PAC discharge. Patients are excluded if they are: homeless or incarcerated; do not have a working telephone; reside in long-term care prior to hospitalization; have a limited (< 6 months) life expectancy per medical record documentation (e.g., stage 4 metastatic cancer diagnosis, hospice referral); currently enrolled in a drug trial; or, expected to discharge from the hospital in less than 48 h (due to inadequate time for study assessments). Lastly, patients must be able to speak English and have the capacity to provide self-consent or have a surrogate (i.e., family member or friend) willing to consent on their behalf.

### Randomization, allocation, and study phases

Trained research personnel approach eligible patients at the bedside during their hospital stay to obtain informed, written consent (Fig. 1. Phase 1). The consent process also includes a release of healthcare information to allow access to participants’ health records at VUMC or elsewhere for emergency room visits or hospitalizations during the study period.

Following consent, research personnel complete a baseline interview, and participants are randomized to the intervention or control groups (Fig. 1. Phase 2). Participants are randomized in a permuted block fashion, in randomly selected block sizes of two or four. The complete randomization table was created and uploaded to the VUMC Research Electronic Data Capture (REDCap) system, which automatically assigns the randomized allocations to authorized users [68].

Those randomized to intervention receive a second interview with a study Pharmacist (PharmD) or Nurse Practitioner (NP) to review deprescribing recommendations, assess their preferences related to medication changes, and communicate with the treatment team (described below). Participants in both groups are followed through their PAC stay (Fig. 1. Phase 3) during which intervention-initiated deprescribing continues for those in the intervention group. After PAC discharge, research personnel conduct a follow-up telephone interview at 7 (range 4–10) and 60 (range 50–70) days and a home visit at 90 (range 76–104) days (Fig. 1. Phase 4). Additional data are obtained from the hospital medical record, PAC medical record and pharmacy records.

### Treatments

#### Intervention: Patient-centered Deprescribing conceptual framework

**Fig. 2:**
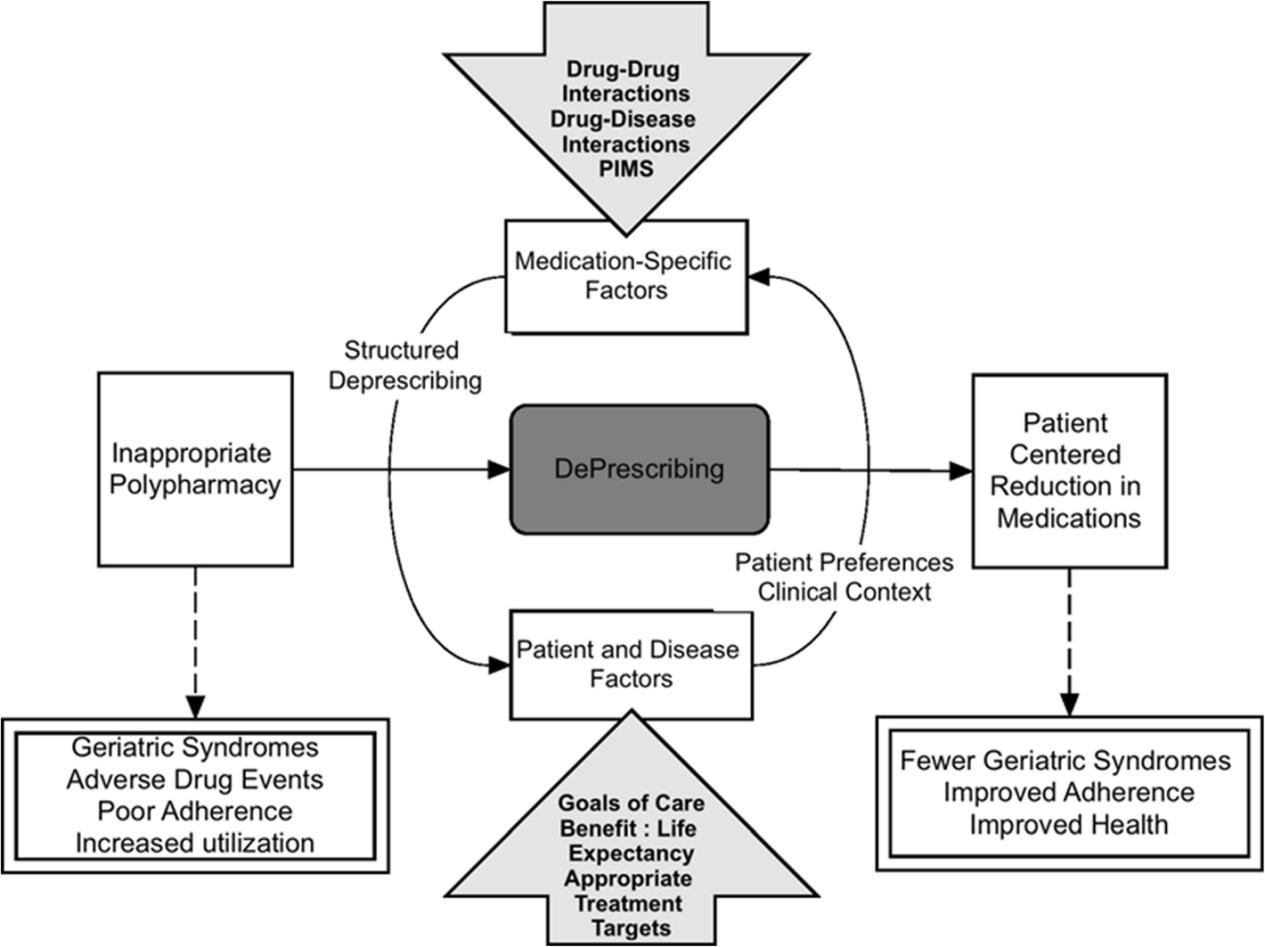
Conceptual Framework for Deprescribing Intervention (Shed-MEDS)

The Shed-MEDS deprescribing protocol is based on a conceptual framework that considers patient and disease factors, life expectancy, goals of care, appropriate treatment targets and the duration of treatment required for benefit (Fig. 2) [17]. Medication-specific factors are also incorporated for minimizing inappropriate medication use, such as drug-specific safety profiles, drug-drug and drug-disease interactions [69]. Finally, patient preferences are viewed as a key component that informs final deprescribing actions by identifying medications the patient is willing to deprescribe (e.g., due to lack of efficacy, poor compliance, side effects or cost burden) and potential barriers to deprescribing (e.g., concerns about worsening of symptoms). Specifically, our goal is to identify opportunities for deprescribing wherein clinical evidence aligns with patient preferences. Importantly, deprescribing is defined as stopping medications or reducing the dose or frequency of administration. All medications (prescription and over-the-counter medications including vitamins and herbal supplements) are reviewed for deprescribing potential.

#### Intervention: Patient-centered Deprescribing steps

Following the baseline patient/surrogate interview and comprehensive medication history for both intervention and control groups, a study PharmD or NP reviews the reconciled medication list for intervention participants only. For each medication on the list, the PharmD or NP conducts a medical record review to determine the medication-indication pairing and deprescribing rationale. The indication (i.e. diagnosis or symptom) for each prescribed medication is specified; if an indication does not exist, “no indication” or “indication unknown” is recorded. Rationales for deprescribing, including no indication for medication, are selected from the list in Table 2. Multiple rationales may be applicable to an individual medication.

**Table 2:** Deprescribing Rationales

A. No indication for medication / Indication not clear B. Absolute contraindication C. Wrong dose or directions for medication D. Inappropriate for current indication due to: 1. Indication has resolved 2. Patient is below treatment threshold 3. Treating guidelines have changed, medication no longer indicated 4. Wrong indication for medication E. Medication is ineffective as evidenced by no change in symptom or condition F. Duplicate medication for same indication	G. High risk medication based on: 1. Potential drug-drug interaction 2. Potential drug-disease interaction (e.g. associated with geriatric syndrome) 3. On explicit list of PIMs (Beer’s, STOPP and/or RASP) H. Medications are inconsistent with goals of care H. Risk > benefit given patients limited life expectancy I. Evidence of poor adherence or high risk of poor adherence (directions impractical, high cost) J. Medication currently indicated, but is time-limited and indication expected to resolve

For each medication recommended for deprescribing, the deprescribing action is specified as follows: (1) stop prior to hospital discharge without the need for monitoring; (2) stop prior to hospital discharge with symptom/physiologic monitoring; (3) stop at specified time point following hospital discharge; (4) reduce over time with monitoring until medication is stopped; (5) reduce to lower dose without the need for monitoring; and. (6) reduce to lower dose with symptom /physiologic monitoring. The advantage of initiating deprescribing actions during the hospital stay for patients being discharged to a PAC facility (SNF or IPR) is that the SNF/IPR setting affords the opportunity for continued symptom/physiologic monitoring and additional dose reductions or titrations.

Once deprescribing recommendations have been established based on medical record review, the study PharmD/NP conducts a structured interview with the patient/surrogate to elicit their medication preferences. The following is assessed for each medication targeted for deprescribing: medication adherence, side effects, perceived benefit (or harm), cost and level of interest in stopping or reducing the medication. Additionally, patients/surrogates are asked if they are interested in stopping or reducing any medication that has not been recommended by the study PharmD/NP. The goal is to involve the patient/surrogate in the decision-making process related to deprescribing to increase the likelihood of adherence following hospital and PAC discharge.

The agreed-upon list of deprescribing actions is then communicated to the hospital treatment team and/or primary outpatient prescriber(s) via electronic health record messaging (if a VUMC provider) or telephone (if a non-VUMC provider). The goal of these conversations is to obtain provider feedback about the proposed medication changes and facilitate medication updates in the patient’s medical record(s). The study PharmD/NP coordinates with the hospital treatment team to incorporate all final deprescribing actions into the transfer orders at the time of hospital discharge to the PAC facility.

To ensure understanding, the study PharmD/NP contacts a designated PAC provider via telephone within 24 h of transfer to review the orders, with particular attention to medications that have a dose reduction, titration and/or the need for symptom/physiologic monitoring. Additionally, medications that were stopped prior to hospital discharge and the deprescribing rationale are reviewed during this call. The study PharmD/NP-to-PAC nurse phone call is also used to inform the PAC facility of amended orders if the patient discharges to the PAC facility before deprescribing actions can be incorporated into the initial transfer orders. Additionally, a copy of the amended orders is sent to the PAC facility.

Following the initial PAC hand-off, the study PharmD/NP initiates a weekly conversation via telephone with a designated PAC provider to review intervention participants’ medication administration record and evidence of symptom worsening or drug withdrawal. Reasons for modifying the transfer medication list during the PAC stay are discussed and intervened upon, as necessary. The study PharmD/NP also may continue to communicate with the original prescriber (primary care provider or specialist) during the patient’s PAC stay to confirm their agreement with continued medication changes. At the time of discharge from the PAC facility, a final medication list is reconciled by the study team for intervention participants. This list is provided to the patient’s primary outpatient prescriber(s), along with suggestions for continued medication management and deprescribing, when applicable, along with the rationale for these changes.

#### Control group: Usual care

Patients randomized to usual care undergo medication review performed by the study team at hospital admission, hospital discharge, and PAC discharge. The goal of this medication review process is to obtain an accurate medication list for study records and assessment without making recommendations about deprescribing. We chose a usual care comparator, as there are no current established gold-standard, effective, deprescribing interventions. All medication decisions for the control group, prescribing or deprescribing, are at the discretion of the treating teams in the hospital, PAC setting, and post-discharge providers. The study team alerts provider(s) to substantial medication discrepancies that pose imminent safety hazards discovered during the medication history-taking process.

#### Interview measures and data collection

Table 1 shows all study measures and the data collection time points. To promote participant retention and complete follow-up, participants are paid $10 after baseline assessments are complete and up to an additional $40 for completing follow-up time points (7, 60 and 90 days). Participants may be discontinued from the study for the following reasons, in which case there are no data collected past the date of discontinuation: discharge from the hospital to a non-partner PAC facility or home, consent withdrawal, transition to hospice care or death. Each study measure listed in Table 1 is briefly described below.

#### Demographic, comorbidity, and attitudes toward Deprescribing

Research personnel use a standardized form to abstract information from participants’ electronic medical record upon enrollment to include demographics (age, gender, race/ethnicity), contact information (home address/telephone), insurance status, education level, outpatient providers and pharmacies. We also abstract the participant’s location immediately prior to hospital admission, hospital admission and discharge dates (length of stay), admission diagnoses, hospital service, pre-admission and in-hospital medication lists. These data are reviewed and verified with the patient and/or surrogate during a baseline interview at the hospital bedside (Fig. 1. Phase 1).

Medical diagnoses are used to calculate the Charlson Comorbidity Score [70] for each patient, which ranges from 0 to 31, with a higher score indicating more comorbid illness. There is an additional one-unit increase in the weighted score for every decade starting from age 50. Data sources for comorbidities are the ICD-10 diagnostic criteria from the last 12 months [71]. Medical record data also is used to calculate the Walter Index, which is a prognostic tool to predict one-year mortality among older adults after hospital discharge [72]. A higher score is indicative of a greater likelihood of mortality within one year (e.g., scores > 6 = 64% estimated mortality rate). Lastly, medical diagnoses and history are used to calculate the older patient’s risk for an adverse drug event via the GerontoNet Adverse Drug Event risk assessment [20].

We administer the Patients’ Attitudes Toward Deprescribing (PATD) tool, which consists of 15 items. Ten items are statements with a 5-point Likert scale response option from “strongly agree” to “strongly disagree”. Examples include: “I feel that I am taking a large number of medicines” and “I believe that all my medicines are necessary”. The remaining five questions are related to the patient’s perception of their total number of medications (e.g., “How many different tablets/capsules per day would you consider to be a lot?”), history and comfort level with stopping one or more medicines [73].

#### Primary outcome measure: Medications

Multiple data sources are used to compile a comprehensive list of all medications to include the hospital medical record, patient/surrogate interview, pharmacy records (refill history) and the PAC (SNF or IPR) medical record. Medications at study enrollment (Fig. 1. Phase 1. Baseline) include any medication that has the potential to be continued at the time of hospital discharge. This includes (a) pre-hospital medications, (b) active in-hospital medications not on the pre-hospital medication list; and, (c) medications identified via patient/surrogate interview and/or pharmacy records, including mail-order pharmacies, for the three months prior to hospitalization. All prescribed (scheduled and as needed) and over-the-counter medications (including vitamins and herbal supplements) administered by any route other than topical are included in the patient’s comprehensive medication list.

Potentially inappropriate medications (PIMs) are defined by previously published lists including the recently updated Beers criteria [12], the STOPP criteria [13, 14], and the Rationalization of home medication by an Adjusted STOPP in older Patients (RASP) list [15], for which there is a large degree of overlap. The total number of PIMs is the sum of unique medications found on any of these explicit lists. The total number of medications associated with geriatric syndromes is based on a detailed list of specific medications delineated in a prior study [45]. These medications have an evidence-base, expert consensus or care practice guideline, and/or > 5% side effect incidence per the Lexicomp Online® database and/or Food and Drug Administration (FDA) approved package inserts indicating an association between the medication and one or more geriatric syndromes.

Based on the comprehensive list of all medications, a Drug Burden Index (DBI) score is calculated per participant for anticholinergic (DBI_AC_) and sedative medications (DBI_S_) separately. Anticholinergic and sedative medications have been strongly linked to functional impairment [25, 26, 34, 74], falls [75–77], and delirium [78–80]. The DBI is the sum of each individual anticholinergic/sedative medication’s prescribed daily dose divided by the sum of the minimum effective dose (as estimated by the FDA minimum recommended dose) and the patient’s daily dose.

#### Secondary outcome measures: Geriatric syndromes

Table 1 lists eight geriatric syndromes, each of which is assessed by trained research personnel via a standardized instrument and patient interview during their hospital stay (Fig. 1. Phase 1) and again following PAC discharge (Fig. 1. Phase 4) via telephone at 7 days and in-person during a home visit at 90 days. Additionally, a copy of the discharge Minimum Data Set (MDS) assessment or Inpatient Rehabilitation Facility-Patient Assessment Instrument (IRF-PAI), which is required for all SNF or IPR patients respectively, is retrieved from the PAC medical record for each participant as a measure of these syndromes during the PAC stay.

The Brief Confusion Assessment Method (bCAM) is a screening tool for delirium that has been validated among hospitalized, older patients [81, 82]. If the patient screens positive for delirium, no other patient interviews are conducted at that time. Research personnel continue to re-assess delirium daily during hospitalization; and, when the patient screens negative for delirium via the bCAM, the remaining assessments are administered to the patient. If delirium continues throughout the hospital stay or the patient is otherwise unable or unwilling to complete the assessments, the surrogate is approached for a sub-set of the geriatric syndrome assessments (i.e., incontinence, nutrition and fall history). The bCAM is not repeated by research personnel at 7-day follow-up because it has limited validity when administered via the telephone [83]; thus, it is repeated only at 90-day follow-up during the in-person home visit (Table 1). Additionally, PAC personnel use the CAM (i.e., the Confusion Assessment Method, [CAM]), which is a modified version of the bCAM, to assess delirium during the PAC stay via the MDS assessment.

Cognitive impairment is assessed with the Brief Interview for Mental Status (BIMS), which has a total score range from 0 to 15 (0–7: severe impairment; 8–12: moderate impairment; 13–15: cognitively intact) [84, 85]. The Patient Health Questionnaire (PHQ-9) is a validated tool to assess depression symptoms and severity [86]. Each item is scored from 0 (“not at all”) to 3 (“nearly every day”) to yield a total score range from 0 (no depressive symptoms) to 27 (severe depression). Both the BIMS and the PHQ-9 are also part of the MDS assessment.

The International Consultation on Incontinence Questionnaire - Urinary Incontinence Short Form (*ICIQ-UI SF*) consists of four items that assess the symptoms, frequency and impact of urinary incontinence on quality of life. [87] This tool has well established reliability (Cronbach’s alpha = .95) and validity, including sensitivity to treatment. The MDS has one item for urinary incontinence frequency scored 0 (‘always continent’) to 3 ‘always incontinent’).

Nutritional risk is assessed with the 10-item “Determine Your Nutritional Health checklist”, which yields a total nutritional risk score of 0–2 (“low”), 3–5 (“moderate”), or > 6 (“high”). The DETERMINE checklist has been validated in a longitudinal study of community-dwelling older adults [88]. If the participant endorses a weight change of 10 pounds or more on the DETERMINE item, a structured follow-up question is posed to clarify whether the weight change reflects a gain versus a loss. The MDS has one item for unintentional weight loss, defined as 5% or more in 30 days or 10% or more in 180 days.

Pain is assessed with the Brief Pain Inventory (BPI) Short-Form, which is a validated instrument for assessing pain location, severity and interference with daily activities among older adults [89, 90]. Pain severity is based on four questions wherein participants use a 0 (no pain) to 10 (worse pain imaginable) scale to rate their pain in the last 24-h under four conditions: at its worst, at its least, on average, and now. Pain is assessed in the PAC setting via the MDS, which includes items related to pain frequency, effect on function and intensity.

Pressure ulcers (number and stages 1–4 or unstageable) are abstracted from the hospital and PAC medical record and confirmed via patient/surrogate interview at each time point. Fall history in the month prior to hospitalization (frequency = 0, 1 or 2 or more fall events) is assessed via patient/surrogate interview. Falls during the PAC stay are abstracted from the PAC medical record (number of falls since PAC admission) and confirmed via patient/surrogate interview. Additionally, patients are interviewed about falls after PAC discharge during the follow-up phase.

#### Secondary outcomes: Medication adherence and functional health status

The Adherence to Refills and Medications Scale (ARMS) consists of 12 items to assess overall medication adherence, with a total score range of 12 to 48. A lower score is indicative of better medication adherence [91]. Example questions include: “How often do you forget to take your medicines?”, “How often do you miss taking your medicines when you feel better?”, and “How often do you put off refilling your medicines because they cost too much money?” Response options are on a 4-point Likert scale that ranges from “none of the time” to “all of the time”. The Vulnerable Elders Survey (VES-13) is a functional measure of health status that assesses a patient’s cognitive, physical and self-care activities and includes an item for self-rated health status. Scores range from 1 to 10, with a lower score indicative of better health [92, 93].

#### Safety measures: Healthcare utilization and adverse drug events

Unplanned healthcare utilization (intensive care unit transfers, emergency department visit and/or hospitalizations) is monitored throughout all study phases for each participant. Unplanned events are assessed by physician co-investigators blind to group assignment using a structured review protocol of all relevant medical records from both within and outside of VUMC. Physician reviewers determine whether the unplanned healthcare utilization is related to an adverse drug event (ADE) and medication withdrawal (i.e., adverse drug withdrawal event, [ADWE]) using the 10-question Drug Withdrawal Probability Scale [94], a scale based on the Naranjo algorithm [95], which is a validated scoring system to assess causality of adverse drug events. Deaths and transitions to hospice are also monitored for all participants.

### Blinding

Due to the nature of the intervention, participants are not blinded to the intervention. In addition, the PharmD and NP delivering the patient-centered deprescribing intervention cannot be blinded. However, the investigators and the statistician performing data analyses are blinded to group assignment. Additionally, the physician co-investigators who review patients’ medical records to assess ADEs and ADWEs (safety measures) are blinded to group assignment.

### Statistical methods

The effect of the intervention on the total number of medications, PIMs, and medications associated with geriatric syndromes (MAGS) at hospital discharge, PAC discharge, and 90-days following PAC discharge will be quantified using mixed effects Poisson regression, allowing for over dispersion (more or less variability than is expected under the Poisson regression model), and adjusting for measurement time point (as a categorical covariate), the total number of medications at participant enrollment and the interaction of intervention and time point. The within-subject correlation among repeated measurements will be modeled using a random intercept term, indexed by subject. The overall statistical significance of the intervention effect will be evaluated using a Wald-type multiple degree-of-freedom test against the null hypothesis of no effect at any time point after randomization. *P*-values less than 0.05 will be considered statistically significant. The intervention effect at each time point will be summarized using a Wald-type 95% confidence interval. Model fit will be assessed by examining the associated Pearson residuals and using other graphical methods. Alternative regression techniques may be used in the case of poor fit, for example, cumulative logit regression methods.

The effect of intervention on the anticholinergic and sedative drug burden (DBI) scores at hospital discharge, PAC discharge and 90-days following PAC discharge will be assessed in a similar fashion as described above, using linear mixed effects regression rather than Poisson regression. Due to the constrained nature of the score values (0–1 for anticholinergic and sedative drug burden scores), an alternative method may be required, such a ‘beta’ regression, which is suitable when such scores frequently occur at a boundary (0 or 1).

The prevalence and severity of geriatric syndromes will be analyzed at each time point following PAC discharge. The effects of intervention on the prevalence of each type of geriatric syndrome will be assessed using mixed-effects logistic regression, in a manner similar to that described above. The severity of each geriatric syndrome is measured on an ordinal scale, where the absence of a geriatric syndrome will be treated as the lowest category of severity. Thus, severity will be similarly analyzed using mixed-effects proportional odds logistic regression.

Patient medication adherence will be measured using the ARMS total score, which is based on an ordinal scale. Functional health status is measured using the VES-13 total score, which is also an ordinal outcome. The effects of intervention on these outcomes at 7 and 90-days following PAC discharge will be quantified using mixed-effects logistic regression or proportional odds logistic regression in a manner that is analogous to that described above.

### Missing data and intent-to-treat

We will examine the incidence of missing data by group. If imbalances are found, we will implement a series of sensitivity analyses, using a chained-equations multiple imputation method, to assess the degree of bias that might be induced by missing data. Records for all randomized patients will be included in analyses.

### Preservation of type-I error rate

Overall effectiveness of the intervention will be assessed using a multiple degree-of-freedom test against the null hypothesis of no intervention effect on the primary outcome (change in the total number of medications) at any time point after randomization. A *p*-value less than 0.05 will be considered statistically significant. Thus, the type-I error rate for the assessment of overall effectiveness is fixed at 5%. All other outcomes will be treated as secondary or exploratory endpoints, or as components of the primary outcome (e.g., PIMS, MAGS, DBI). Statistical tests and confidence intervals will be used to summarize the effect of intervention on secondary/exploratory outcomes but will not be used to assess overall effectiveness. We will not make adjustments to control the familywise type-I error potentially associated with tests of secondary / exploratory outcomes [96].

### Power and sample size

The overall effectiveness of the intervention will be evaluated based on the primary outcome measure, which is total medication count. Based on our current enrollment rate, we estimate that approximately 144 patients per year will enroll in the study, or 576 across all four years of enrollment. Among those, we estimate that 27.5% will either die or otherwise be lost from the study prior to the 90-day post PAC discharge follow up period. Thus, across all four years of enrollment, an estimated 420 patients will contribute measurements at 90 days. Although 567 is the expected total enrollment, we conservatively use 420 to estimate the statistical power associated with the assessment of overall effectiveness (i.e., overall completion rate) rather than enrollment rate, to account for estimated attrition across all study phases.

Preliminary data is available for the effect of deprescribing on the reduction in the counts of total medications [97]. Our pilot intervention (*N* = 40) was associated with roughly a 50% reduction in the count of total medications from enrollment to hospital discharge, whereas a roughly 25% reduction was observed in the control group receiving routine hospital care [97]. Using these preliminary data and mixed-effects Poisson regression methods, we implemented a simulation-based power analysis wherein we assumed that this effect would be attenuated by 20% at SNF discharge, and again by 20% at the 90-day follow up. Using a sample size of 420, there is greater than 95% power to detect a 50% versus 25% reduction in total medications. There is approximately 90% power to detect a 30% versus 25% reduction in total medications, and 80% power to detect a 27.5% versus 25% reduction in total medications. Thus, the target sample size provides some protection against effects that are substantially smaller than that observed in our preliminary data.

### Data integrity and privacy

All study data are collected by trained research personnel during each study phase. Participants each receive a unique study identifier. All data are collected via hard copy forms and managed using the Research Electronic Data Capture (REDCap) platform [67], Microsoft Excel and SPSS (Version 25). REDCap is a secure, web-based application designed to support data entry, validation and management. All hard copy data collection forms are kept in locked, secure file cabinets and other electronic data is stored on a secure shared computer drive, which is only accessible by study personnel. Study coordinators conduct weekly quality assurance reviews to ensure data accuracy when data is translated from hard copy forms to electronic databases.

### Access to data and dissemination policy

Trial investigators will have full access to the final trial dataset. There are no contractual agreements that limit such access to investigators. Investigators plan on publishing results for all pre-specified primary and secondary outcomes in the peer-reviewed literature. Publications will include publication of the full study protocol and access to statistical code, upon request for review.

### Data and safety monitoring board (DSMB)

This clinical trial has an DSMB. The DSMB is independent, and acts in an advisory capacity to the NIA Director to monitor participant safety, data quality and evaluate the progress of the study. The DSMB consists of five members, and 3 members constitute a quorum. Members were selected by an NIA Program Official in consultation with the investigators, and the NIA Director approved the composition of the DSMB and its membership. The DSMB includes experts in the fields of relevant clinical expertise in geriatrics, clinical trial methodology, and biostatistics. Monthly safety reports are submitted to NIA and semi-annual DSMB meetings are held to review safety data. The NIA Program Official or designee attend each meeting. An emergency meeting of the DSMB may be called at any time by the Chair or by the NIA should participant safety questions or other unanticipated problems arise. In the case of a serious adverse event that results in death, the Principal Investigators are required to inform the NIA within 48 h of notification.

## Discussion

Polypharmacy is prevalent among hospitalized older patients; however, the health outcomes associated with deprescribing are largely unknown, particularly as these relate to geriatric syndromes. The VUMC Shed-MEDS study is the first randomized controlled trial to evaluate a comprehensive deprescribing intervention for hospitalized, older patients discharged to a PAC facility (SNF or IPR). It is also one of the few deprescribing studies to incorporate patients’ preferences into the decision-making process in a structured way. The results of this trial will quantify the number and type of medication changes that can be initiated and maintained across the continuum of care from hospital to PAC facility to home. Additionally, this trial will examine the impact of medication reductions on adherence, geriatric syndromes and functional health status.

One limitation of the study is the lack of blinding of the patient to the intervention, which could potentially bias the self-reported outcome measures (secondary aim). However, there are measures in place to reduce this bias, including the blinding of outcome assessors and the analytic team to group allocation and the use of standardized, reliable assessment tools for each measure. This study is currently the largest known acute care deprescribing trial, to date, and the first to follow patients into the PAC setting, generating substantial new knowledge related to the efficacy and safety of acute care-initiated patient-centered deprescribing efforts for older patients with polypharmacy.

## Trial status

### Enrollment to date

Trial recruitment began March 1st, 2017. The trial protocol has undergone revisions and is currently at version two. We anticipate that recruitment will be completed October 31, 2020.

## Additional file

Additional file 1: SPIRIT Checklist (DOCX 48 kb)

## Abbreviations

ADE: Adverse Drug Event
ADWE: Adverse Drug Withdrawal Event
ARMS: Adherence to Refills and Medications Scale
bCAM: Brief Confusion Assessment Method
BIMS: Brief Interview for Mental Status
BPI: Brief Pain Inventory
CAM: Confusion Assessment Method
DBI: Drug Burden Index
DETERMINE: Determine Your Nutritional Health Checklist
DSMB: Data Safety and Monitoring Board
FDA: Food and Drug Administration
ICIQ-UI SF: International Consultation on Incontinence Questionnaire - Urinary Incontinence Short Form
IPR: Inpatient Rehabilitation Facility
MDS: Minimum Data Set
NIA: National Institute on Aging
PAC: Post-acute Care
PATD: Patients’ Attitudes Toward Deprescribing
PHQ: The Patient Health Questionnaire
PIMS: Potentially inappropriate medications
RASP: Rationalization of home medication by an Adjusted STOPP in older Patients
SNF: Skilled Nursing Facility
STOPP: Screening Tool of Older Persons’ potentially inappropriate Prescriptions
VES: Vulnerable Elders Survey
VUMC: Vanderbilt University Medical Center
